# Systematic Innominate Artery Cannulation Strategy in Acute Type A Aortic Dissection: Better Perfusion, Better Results

**DOI:** 10.3390/jcm12082851

**Published:** 2023-04-13

**Authors:** Horea Feier, Andrei Grigorescu, Laurentiu Braescu, Lucian Falnita, Marius Sintean, Constantin Tudor Luca, Mihaela Mocan

**Affiliations:** 1 Institute for Cardiovascular Diseases, 300310 Timisoara, Romania; 2 Department of Cardiology, University of Medicine and Pharmacy, 300041 Timisoara, Romania; 3 Department of Internal Medicine, University of Medicine and Pharmacy Iuliu Hatieganu, 400012 Cluj-Napoca, Romania

**Keywords:** aorta, dissection, cannulation, malperfusion

## Abstract

(1) Background: Arterial cannulation in type A acute aortic dissection (TAAAD) is still subject to debate. We describe a systematic approach of using the innominate artery for arterial perfusion (2) Methods: The hospital records of 110 consecutive patients with acute TAAAD operated on between January 2014 and December 2022 were retrospectively analyzed. The effect of the cannulation site on early and late mortality, as well as on cardio-pulmonary perfusion indices (lactate and base excess levels, and cooling and rewarming speed) were investigated. (3) Results: There was a significant difference in early mortality (8.82% vs. 40.79%, *p* < 0.01) but no difference in long-term survival beyond the first 30 days. Using the innominate artery enabled the use of approximately 20% higher CPB flows (2.73 ± 0.1 vs. 2.42 ± 0.06 L/min/m^2^ BSA, *p* < 0.01), which resulted in more rapid cooling (1.89 ± 0.77 vs. 3.13 ± 1.62 min/°C/m^2^ BSA, *p* < 0.01), rewarming (2.84 ± 1.36 vs. 4.22 ± 2.23, *p* < 0.01), lower mean base excess levels during CPB (−5.01 ± 2.99 mEq/L vs. −6.66 ± 3.37 mEq/L, *p* = 0.01) and lower lactate levels at the end of the procedure (4.02 ± 2.48 mmol/L vs. 6.63 ± 4.17 mmol/L, *p* < 0.01). Postoperative permanent neurologic insult (3.12% vs. 20%, *p* = 0.02) and acute kidney injury (3.12% vs. 32.81%, *p* < 0.01) were significantly reduced. (4) Conclusions: systematic use of the innominate artery enables better perfusion and superior results in TAAAD repair.

## 1. Introduction

Acute type A aortic dissection (TAAAD) consistently ranks among the deadliest cardiovascular pathologies, with early mortality between 15–30% [1,2,3], even in contemporary, large-scale databases, such as the Society of Thoracic Surgeons’ Adult Cardiac Surgery Database [4], but there is a constant trend to improve results in high-volume centers, as reported in the International Registry of Aortic Dissections (IRAD) [5].

Mortality in unoperated patients has been found to be as high as 1% per hour in historic studies such as that by Hirst et al. in the 1950s [6], but has fallen in recent studies to 0.5% per hour [5]. It can result from cardiac tamponade, aortic rupture, low cardiac output or malperfusion. Treatment involves the resection of the ascending aorta and variable parts of the aortic arch under hypothermic cardiopulmonary bypass.

Establishing cardiopulmonary bypass in TAAAD has been traditionally performed by femoral canulation [7], but antegrade perfusion techniques such as axillary artery [8], direct aortic [9] or even transapical [10] cannulation are preferred for their lower stroke risk [4]. Di Eusanio has been the first to propose the use of the innominate artery as an arterial cannulation site in surgery of the aorta [11], but he used it only in 2 out of 55 patients with TAAAD in his initial study. Indeed, the use of the innominate artery in TAAAD is reported in a minority of cases, even in recent papers, as concern has been raised over using a potentially dissected vessel, located in close vicinity to the aortic arch, for arterial inflow [12,13,14].

We present our experience with systematically using the innominate artery in TAAAD during a 5-year time-frame.

## 2. Experimental Section

All admissions for TAAAD in our institution between January 2014 and December 2022 were retrospectively analyzed. TAAAD was defined as an aortic dissection diagnosed less than 14 days after the onset of symptoms. During this period, 110 consecutive patients were admitted and operated upon with the above diagnosis.

Patients were diagnosed on the basis of a contrast-enhanced chest CT scan and/or transthoracic ultrasonography. Upon admission, we performed transthoracic echocardiography in all patients and took venous blood samples for complete analysis.

### 2.1. Surgical Technique

Stable patients were peripherally cannulated prior to sternotomy using the axillary or femoral arteries. In unstable patients or when using the innominate artery for arterial inflow, we performed sternotomy and a limited pericardotomy first in order to relieve cardiac tamponade, and performed cannulation afterwards. Direct cannulation was used for the femoral artery, whereas the axillary artery was used after sewing an 8 mm Dacron graft in an end-to-side fashion at its level.

We used the innominate artery but also the other arterial sites, at the beginning of our experience; nevertheless, the innominate artery was used exclusively from 2018 onwards. The artery was prepared from its origin until its bifurcation, corresponding to the right sternoclavicular joint. Its state was assessed before the procedure on the contrast-enhanced CT-scans of the patient. If the origin of the innominate artery was not severely dissected, direct cannulation was undertaken using a double 4-0 polypropylene purse-string and a right-angled 22/24 Fr aortic cannula (Medtronic DLP), using transversal arteriotomy.

**Figure 1:**
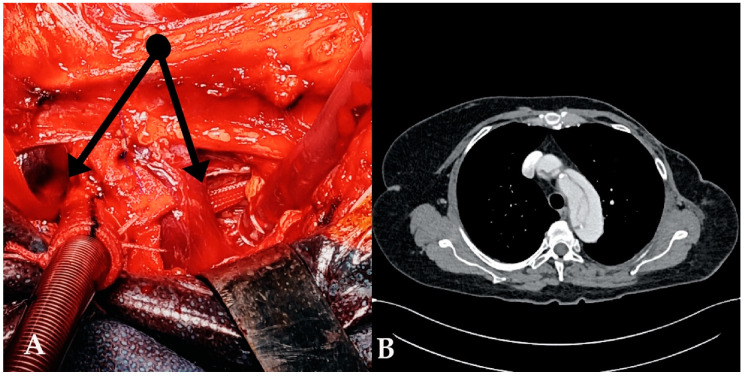
Cannulation of innominate artery ((**A**), arrows) in extreme case with completely compressed true lumen. ((**B**), see Video S1). An 8 mm Dacron graft was anastomosed to the medial side of the artery using a Teflon felt-reinforced polypropylene 5-0 suture.

If the artery was extensively dissected, 5000 UI of heparin was given intravenously, the artery was clamped using two separate clamps and a longitudinal arteriotomy was performed. The presence of a dissection flap at this level was assessed and if confirmed, an 8 mm Dacron graft was anastomosed to the true lumen of the artery using polypropylene 5-0 and an outer Teflon band (Figure 1).

Heparinization was completed after unclamping the anastomosis. Cannulation was systematically undertaken after 2018, even in extreme cases, in which the true lumen of the artery was completely compressed by the false lumen and the patient had ischemia of the right arm (see Video S1).

Venous inflow was obtained via the right atrium in all patients. The left side of the heart was vented using the right superior pulmonary vein. The patient was cooled to a core temperature of 25 degrees Celsius. The aorta was cross-clamped just before reaching this temperature and transected, and a dose of cardioplegia was delivered via the coronary ostia. The ascending aorta was inspected for the entry-site of the dissection. Distal anastomosis was performed in an open fashion. Cerebral protection was achieved using antegrade cerebral perfusion at a rate of 10 mL/kg/min by clamping the origin of the innominate artery. After completing distal anastomosis, systemic perfusion was resumed and the patient was rewarmed in an antegrade fashion using either the previously cannulated axillary or innominate artery, a side-branch in the aortic prosthesis or direct cannulation of the Dacron graft. Proximal anastomosis was performed next. The root and aortic valve were assessed. The reasons for performing complete root resection were extensive dissection involving more than the non-coronary sinus, a dilated aortic root (>5 cm) or an abnormal aortic valve. In such cases, the root was replaced using a conduit graft, root reimplantation or remodeling techniques. The aortic root was treated conservatively, in most cases, by reapproximating the dissecting layers with surgical glue and reinforcing them with generous amounts of outer Teflon felts.

### 2.2. Perfusion Indices

Lactate values were measured upon admission and then upon arrival in the ICU. The patient’s base excess levels were measured at 4 time points during the procedure (prior to bypass, during the bypass at 2 time points and again after discontinuing CPB) as well as upon arrival in the ICU. The mean base excess value was computed during the procedure. The lowest central temperature level was noted and the time-frame from beginning of CPB until this temperature was reached was measured, as was the time needed to rewarm the patient to 37 degrees Celsius.

### 2.3. Statistical Analysis

Continuous variables were expressed as mean ± SD. Categorical variables were presented as percentages. Student’s t-test with or without Satterwaithe’s correction was used on normally distributed continuous variables. Mann–Whitney’s rank sum test was used for the other continuous variables. For categorical variables, we employed the chi-squared test. Univariate analyses were performed in order to determine the variables associated with early mortality (<30 days). Variables that achieved a *p* value of <0.2 in the univariate analysis were introduced in a logistic regression model with early death as the dependent variable. The operating surgeon as a categorical variable was taken into account. An analysis of covariance was performed in order to account for baseline differences between the groups with respect to metabolic variables. A survival analysis was performed on the late (>30 days) hazard phase using log-rank to compare survival between the groups. Kaplan–Meier curves were computed. In all cases, a *p* value of <0.05 was deemed statistically significant.

## 3. Results

There were 76 males (69.09%) and 34 females (30.91%) in our cohort. The mean age was 57.4 ± 11.5 years (range 25–82). Risk factors included hypertension (81%), diabetes (9%), a bicuspid aortic valve (4.55%) and obesity (28.18%). Four cases (3.64%) occurred after the completion of previous cardiac surgical procedures. Four patients had Marfan syndrome (3.64%). The time from the onset of symptoms was 39.12 ± 62.03 h (Table 1).

A third of the patients (32.73%) had a pericardic effusion of ≥10 mm upon preoperative transthoracic ultrasonography. Severe aortic incompetence was found in 42.73% of the patients. The size of the ascending aorta was 5.1 ± 0.98 cm. The mean ejection fraction was 52.18 ± 5.97%. Nineteen patients (17.27%) had branch malperfusion (Penn Ab status), while sixteen (14.55%) were in cardiac tamponade (Penn Ac status).

The innominate artery was used in 34 patients (30.91% of cases), the femoral artery was used in 27 patients (24.55% of cases), the axillary artery was used in 47 patients (42.73% of cases), while the right carotid artery was used in 2 patients (1.82% of cases). The innominate artery was directly cannulated most of the time (26/34); in the remaining cases (8) a Dacron graft was sewn to the artery as it was completely dissected. The entry site was in the ascending aorta or aortic root in 81 patients (73.64% of cases). It was in the aortic arch in similar proportions in patients with innominate artery cannulation (23.53%) or without it (27.63%, *p* = 0.65). In most situations, we performed ascending aortic replacement with a Dacron graft, with or without replacement of the non-coronary sinus (65.45%). A composite replacement of the aortic root was used in 20 patients (18.18% of cases), while a total replacement of the aortic arch was undertaken in 10 patients (9.09% of cases).

### 3.1. Intraoperative Perfusion Indices

Using the innominate artery for inflow allows the use of higher flows, as it is a larger artery than the other canulation candidate sites and located in the vicinity of the aortic arch. Using the femoral, axilary or carotid arteries on the other hand, can limit inflow, as the required flow can surpass the artery’s ability to sustain it, especially in obese patients. The developed flow when using the innominate artery was 2.73 ± 0.1 L/min/m^2^ BSA, versus that of 2.42 ± 0.06 L/min/m^2^ BSA when using the other cannulation sites (*p* < 0.01). This would, in turn, lead to more rapid cooling and rewarming when using systemic hypothermia: the cooling speed was 1.89 ± 0.77 min/degrees Celsius/m^2^ BSA vs. 3.13 ± 1.62 min/degrees Celsius/m^2^ BSA (*p* < 0.01), whereas the rewarming speed was 2.84 ± 1.36 min/degrees Celsius/m^2^ BSA for patients who had their innominate artery cannulated versus 4.22 ± 2.23 min/degrees Celsius/m^2^ BSA for patients who had more peripheral arterial sites cannulated (*p* < 0.01). This also translates into shorter CPB times: 168.47 ± 59.59 min vs. 205.14 ± 79.62 min (*p* = 0.01); please see Table 2.

### 3.2. Early Results

There were 34 early deaths in our cohort (30.91% of cases). Ten patients (9.09% of cases) died during the procedure by rupture, bleeding or circulatory collapse. Patients in which the innominate artery was used for arterial inflow were significantly less likely to die (8.82% vs. 40.79% of cases, *p* < 0.01). A univariate analysis performed on the preoperative variables found that canulation type (*p* < 0.01), Penn non-Aa status (*p* < 0.001), patient age (*p* < 0.01) and serum creatinine (*p* = 0.02) were significantly linked to early death. The surgeon, as a categorical variable, was not a significant risk factor for early death (*p* = 0.05).

Logistic regression performed with early death as the dependent variable on significant (*p* < 0.05) or marginally significant (*p* < 0.2) variables in the univariate analysis found Penn-nonAa status (OR = 7.10, 95% CI = 2.50–20.15, *p* < 0.01), patient age (OR = 1.05 per year, 95% CI = 1.00–1.11, *p* = 0.02) and not using the innominate artery for arterial inflow (OR = 6.04, 95% CI = 1.46–24.84, *p* = 0.01) as independent predictors of early death (Table 3).

Postoperative complication rates were the same of reduced compared to peripheral cannulation strategies (Table 4). In particular, the new onsets of cerebrovascular accidents or of renal failure were significantly reduced in patients systematically cannulated using the innominate artery. There was a trend of increased sepsis and pulmonary dysfunction in patients with more peripheral cannulation sites cannulated.

### 3.3. Metabolic Variables

**Figure 2:**
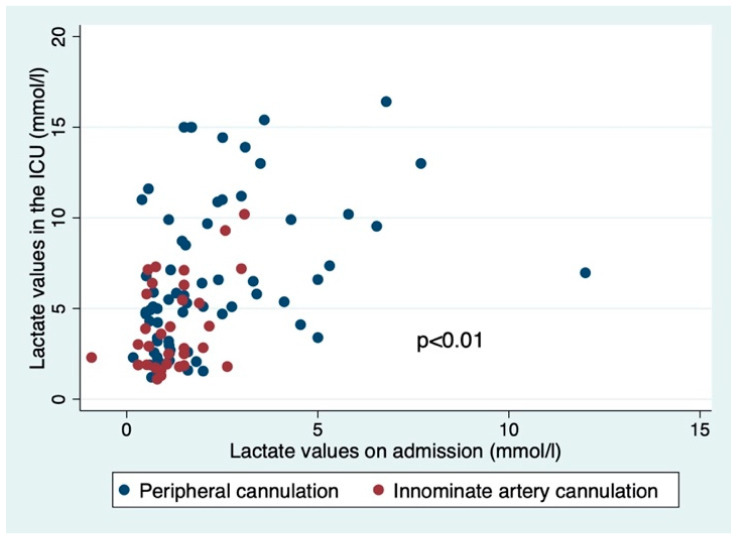
Scatterplot of lactate values in the ICU plotted against lactate values on admission.

The mean base excess values during CPB in patients in whom we used the innominate artery for inflow were smaller (−5.01 ± 2.99 mEq/L vs. −6.66 ± 3.37 mEq/L, *p* = 0.01), which would suggest that better perfusion was enabled by the increased flow rate. The lactate values upon arrival in the ICU in survivors of TAAAD repair were also smaller in those patients where the innominate artery was used for inflow (4.02 ± 2.48 mmol/L vs. 6.63 ± 4.17 mmol/L, *p* < 0.01, Figure 2).

An analysis of covariance with lactate values upon arrival in the ICU as the dependent variable was performed in order to account for baseline differences in lactate values between those two groups. In this analysis, the type of cannulation site used was an independent predictor of lactate values in the ICU (Table 5).

### 3.4. Long-Term Results and Fate of the Innominate Artery

**Figure 3:**
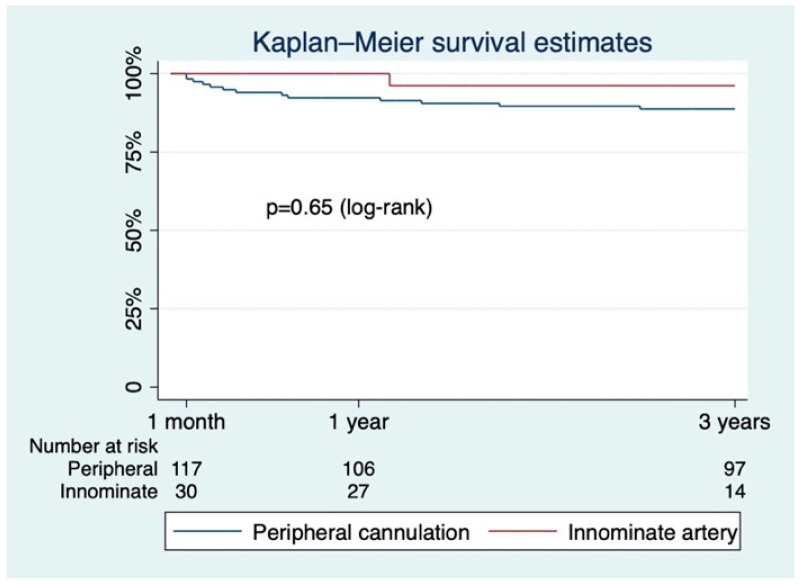
Long-term Kaplan–Meier survival function beyond the acute phase (>30 days).

**Figure 4:**
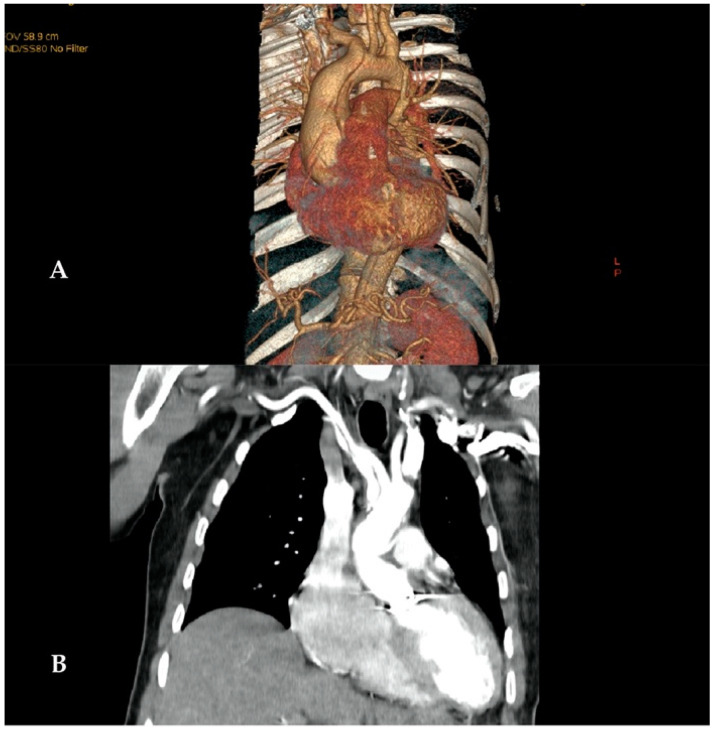
Admission CT scan of a 34-year-old patient with AAAD, anuloaortic ectasia, and a dissection extending well into the innominate artery (**A**). Control CT scan, 6 months after composite replacement of the aortic root and ascending aorta using innominate artery cannulation. The innominate trunk is still dissected with a patent false lumen (**B**).

Survival in the two groups, beyond the first 30 days, was not different at 1 year (95.29 ± 3.25% vs. 100%) and 3 years (95.29 ± 3.25% vs. 96.15 ± 3.77%, *p* = 0.65 log-rank; see Figure 3). The innominate artery would remain dissected, if severely damaged before the procedure, with a patent false lumen after the AAAD repair (Figure 4A,B).

## 4. Discussion

The treatment of TAAAD has undergone refinements that have allowed practitioners to constantly lower surgical mortality; nevertheless, it remains one of the deadliest cardiovascular pathologies. Choosing an appropriate arterial cannulation site is of utmost importance in establishing a CPB that perfuses the true lumen, resolves any pre-existing malperfusion without inducing any new ones and does not cause aortic rupture. The arterial cannulation strategy would also influence the type of cerebral protection employed. Historically, femoral cannulation, away from the dissected aorta, was the preferred strategy. The problems with this attitude would be that arterial flow would be retrograde and as such, pose a higher stroke risk, a risk underscored by several studies [4,15]. Subsequently, a number of different antegrade cannulation techniques have been proposed, such as right axillary artery cannulation, which is perhaps the most widely used at the moment [8], and direct aortic cannulation using the Seldinger technique under echographic epiaortic guidance [9] or even a transapical route [10]. Di Eusanio has proposed the innominate artery as an arterial cannulation site in aortic surgery, but its acceptance in TAAAD has been extremely slow [11]. The reason for this is the proximity of this site to the dissected aorta, which predisposes it to be involved in the dissection process and is the reason for the reluctance to use a dissected artery for CPB. Indeed, in an important study regarding innominate artery cannulation in proximal aortic surgery by Preventza and co-workers of the Texas Heart Institute, the presence of TAAAD was considered a contraindication for innominate artery cannulation [12]. A recent study by the Mayo Group, in which the researchers present their experience in 397 TAAAD surgeries, reports that their preferred arterial cannulation site was the femoral artery (51.5% of cases), followed by the central aortic artery (27.5% of cases), with the innominate artery being used in just 1.3% of cases [16]. Furthermore, even authors that stress their use of the innominate artery in TAAAD are reluctant to use it in the case of a dissected vessel [17]. Fang and associates reported their use of innominate artery cannulation in TAAAD treated by total frozen elephant trunk replacement of the ascending aorta and aortic arch [18]; however, they approached this artery only if it was free of disease.

In contrast to the above reports, this paper presents the systematic use of the innominate artery. Canulation was undertaken with a 20 Fr angled cannula when the dissection flap was not present in the innominate artery. This type of aortic cannula works very well in these situations, as the tip is very short and there is no risk of advancing it in the false lumen of the aortic arch, which would be the case with percutaneous cannulas. Furthermore, it redirects the flow into the aortic arch, to an area of lower resistance. If the artery was dissected, we preferred to sew an 8 mm Dacron prosthesis to the true lumen, reinforcing the arterial wall with outer Teflon strips. This was effective even in extreme situations where the false lumen would completely compress the true lumen and there would be no flow at this level, due to the proximity of the aortic entry site of the dissection to the origin of the artery. In both situations, the closeness of the aortic arch and the size of the innominate artery allowed us to work at approximately 20% higher nominal flows, while using vasodilators to open up the periphery and maximize perfusion. Patients in which the innominate artery was cannulated had consistently lower lactate values upon arrival in the ICU than those in which peripheral vessels were used. There is increased focus on preoperative malperfusion as a significant risk factor for an unfavorable outcome [19,20,21,22], and the ability of the innominate cannulation site to correct it is remarkable.

The fact that we were able to deliver a higher nominal flow also translated to much quicker cooling and rewarming, and a significantly shorter bypass time. This also has a protective effect as the duration of CPB is an established risk factor for early death [23] and acute kidney injury [24] in TAAAD repair. Furthermore, the innominate artery also offers better brain protection and enables the treatment of cerebral malperfusion in patients with TAAAD [25,26]. We can confirm these reports in the present paper as our rate of acute kidney injury and postoperative cerebral insult were significantly reduced in patients in which the innominate artery was used for systemic arterial outflow.

The femoral and axillary arteries are smaller and sensitive to changes in peripheral resistance. This often leads to high pressures on the arterial outflow line of the CPB circuit and the inability to deliver the required flow rate which can result in under-perfused territories or aggravate pre-existing malperfusion. Certain authors supplement axillary cannulation with femoral cannulation or even contralateral axillary cannulation, in order to overcome this problem [27,28]. Direct aortic cannulation offers the same advantages with regard to perfusion but it is much more hazardous, as the risk of selectively perfusing the false lumen is much higher [9], while it is not applicable to patients in cardiogenic shock, or to those with a circumferential entry site [29,30].

There were four Marfan patients in our cohort. Three of them underwent innominate artery cannulation, while in one we used the axillary artery, in the beginning of our experiment. We paid no particular attention to patients with elastopathies (Marfan, Loeys-Dietz, or Ehlers-Danlos Type IV) when choosing the cannulation technique; rather, we paid attention to the extent of the dissection of the artery on the preoperative CT-scan. However, the fragility of the arteries in those patients meant that a direct cannulation technique was preferable.

### Limitations

Our study was retrospective and as such has all the limitations of this type of research. Propensity matching, in order to account for preoperative treatment allocation differences between the groups, could have led to more robust results. Matching results, however, are difficult to obtain in reduced patient groups and, thus, the study required a much larger cohort for meaningful results. Another limitation is that cerebral monitoring was not used in all patients in our cohort. We used near-infrared spectroscopy in our current patients, but this technique is not available for every patient.

## 5. Conclusions

A strategy of systematically using the innominate artery for cannulation is feasible and results in better perfusion, lower postoperative complications and lower mortality in patients with TAAAD. We believe that it should be used in all of such patients.

## Figures and Tables

**Table 1:** Patient population.

Variable	*n* (%)
Male sex	76 (69.09)
Age	57.4 ± 11.5
Redo	4 (3.64)
Acquired risk factors	
Hypertension	89 (80.91)
Diabetes	10 (9.09)
Obesity	31 (28.18)
Congenital risk factors	
Marfan syndrome	4 (3.64)
Anuloaortic ectasia	5 (4.55%)
Presentation	
Shock (Penn Ac)	16 (14.55)
Malperfusion (Penn Ab)	19 (7.27)
Time from onset (hours)	39.12 ± 62.03

**Table 2:** Intraoperative perfusion measurements.

Variable	Innominate Artery	Peripheral Cannulation	*p*
CPB (min)	168.47 ± 59.59	205.14 ± 79.62	<0.01
Cross-clamp time (min)	102.47 ± 37.96	120.93 ± 47.26	0.04
Developed flow (L/min/m^2^ BSA)	2.73 ± 0.1	2.42 ± 0.06	<0.01
Cooling speed (min/°C/m^2^ BSA)	1.89 ± 0.77	3.13 ± 1.62	<0.01
Rewarming speed (min/°C/m^2^ BSA)	2.84 ± 1.36	4.22 ± 2.23	<0.01

**Table 3:** Multivariate risk analysis for early death.

Variable	Odd Ratio	*p*	95% CI	
Penn-nonAa	7.10	<0.01	2.80	25.53
Age	1.05	0.02	1.00	1.11
Canulation Type	6.04	0.01	1.46	24.84
Creatinine	1.17	0.60	0.64	2.14
Ejection fraction	0.96	0.52	0.87	1.06
Entry site	1.59	0.42	0.51	4.95

**Table 4:** Postoperative results.

Variable	Innominate Artery	Peripheral	*p*
Early mortality (<30 days)	3 (8.82%)	31 (40.79%)	<0.01
ICU stay (days, mean ± SD)	7.11 ± 8	4.37 ± 2.64	0.06
Postoperative length of stay (days, mean ± SD)	10.14 ± 4.03	10.97 ± 9.33	0.62
Permanent neurologic injury	1 (3.12%)	13 (20%)	0.02
Acute Kidney failure	1 (3.12%)	21 (32.81%)	<0.01
Sepsis	1 (3.12%)	11 (16.92)	0.05
Pulmonary dysfunction	3 (9.38%)	15 (23.81%)	0.09
Packed red blood cells (units)	2.33 ± 1.79	2.83 ± 2.50	0.30

**Table 5:** Analysis of covariance of lactate values in the ICU.

Variable	Sums of Squares	F Statistic	*p*
Baseline lactate values	183.40	15.29	<0.01
Cannulation type	71.85	5.99	0.01

## Data Availability

The data presented in this study are available on request from the corresponding author. The data are not publicly available due to privacy reasons.

## Supplementary Materials

The following supporting information can be downloaded at: https://www.mdpi.com/article/10.3390/jcm12082851/s1. Video S1: Contrast enhanced chest and abdomen CT scan of a patient with acute Type A aortic dissection. Complete compression of the innominate artery by the false lumen is present, with malperfusion in the right carotid artery. The patient had a 60 mm Hg pressure difference between the right and left arms. Dissection of the left carotid artery was also apparent. The entry site was located at the foot of the innominate artery. The innominate artery was used for cannulation, and the patient had an uneventful recovery.
